# Impact of out-of-home nutrition labelling on people with eating disorders: a systematic review and meta-synthesis

**DOI:** 10.1136/bmjph-2023-000862

**Published:** 2025-01-29

**Authors:** Nora Trompeter, Fiona Duffy, Imogen Peebles, Emily Wadhera, Kate Chambers, Helen Sharpe, Ellen Maloney, Dasha Nicholls, Ulrike Schmidt, Lucy Serpell, Tom Jewell

**Affiliations:** 1 University College London, London, UK; 2 NHS Lothian Child and Adolescent Mental Health Services, Royal Edinburgh Hospital, Edinburgh, UK; 3 Clinical Psychology, University of Edinburgh School of Health in Social Science, Edinburgh, UK; 4 King's College London, London, UK; 5 Department of Brain Sciences, Imperial College London, Harpenden, UK; 6 North East London NHS Foundation Trust, Rainham, UK; 7 Great Ormond Street Hospital for Children NHS Foundation Trust, London, UK

**Keywords:** Public Health, Patient Harm, Primary Prevention, Mental Health

## Abstract

**Objectives:**

Mandatory nutrition labels for out-of-home food consumption have been introduced in several countries to curb rising obesity levels. However, concerns have been raised about the potential negative impacts of such policies on individuals with eating disorders. This review aimed to summarise the literature on the impact of out-of-home nutrition labels on individuals with eating disorders or disordered eating.

**Design:**

A systematic search across eight databases was conducted on 11 October 2023.

**Data sources:**

MEDLINE, EMBASE, APA PsycINFO, Web of Science, ProQuest Dissertations and Theses Global, Scopus and CINAHL. Unpublished studies were searched for on Google Scholar and PsyArXiv.

**Eligibility criteria:**

Studies were included if they assessed the impact of out-of-home nutrition labelling policies on individuals with eating disorders or disordered eating.

**Data extraction and synthesis:**

538 studies were screened, of which 16 studies met inclusion criteria.

**Results:**

The reviewed studies included five experimental/quasi-experimental studies, five cross-sectional studies and six qualitative/mixed-methods studies. Across studies, eating disorder pathology was associated with noticing labels more frequently, paying more attention to caloric intake and more frequent behaviour changes due to caloric values. The metasynthesis identified five themes based on the qualitative findings, *being drawn to calories*, *facilitating the eating disorder*, *reassurance*, *social eating* and *frustration*.

**Conclusions:**

The current review summarised the existing literature on the impact of out-of-home nutrition label policies on individuals with eating disorders. The evidence suggests that there is cause for concern regarding negative impacts, particularly for those with restrictive eating disorders, which should be explored further by research and considered by policymakers when making decisions on public health policies.

**WHAT IS ALREADY KNOWN ON THIS TOPIC**

- Several countries have introduced nutrition labels for out-of-home food consumption.

**WHAT THIS STUDY ADDS**

- This review is the first study to systematically consolidate existing evidence on how out-of-home nutritional labels impact people with lived experience of eating disorders or disordered eating.
- Findings were mixed, but mostly showed negative impacts of out-of-home nutrition labels on people with lived experience of eating disorders or disordered eating.

**HOW THIS STUDY MIGHT AFFECT RESEARCH, PRACTICE OR POLICY**

- This study underscores the importance of investigating the potential impacts of nutrition labelling policies on eating psychopathology.
- Policymakers should consider impacts both on obesity and eating disorders when making decisions about nutrition labelling policies.

Recent efforts to reduce rising obesity rates globally have seen the introduction of several public health policies to reduce individuals’ calorie intake.^1^ Most prominently, several countries have introduced mandatory nutrition labels for out-of-home food consumption (eg, at restaurants). For example, Ontario introduced mandatory calorie labels for out-of-home food and drinks in January 2017 for businesses with 20 or more locations,^2^ and England introduced a similar policy in April 2022 for all businesses with >250 staff.^3^ While designed to reduce the incidence of obesity, concerns have been raised about the potential negative impacts such policies could have on individuals with eating disorders.^4^,^6^ In particular, concerns have been raised that nutrition labels focused on calorie content exacerbate eating disorder cognitions and behaviours that centre around reducing calorie intake, promote preoccupation with calories and increase anxiety when eating out among people with eating disorders. Eating disorders are psychiatric disorders characterised by disturbances in eating behaviours and often accompanied by severe body image concerns.^7^ Eating disorders are heterogeneous and encompass a range of disorders, including anorexia nervosa, bulimia nervosa and binge eating disorder. They are associated with reduced quality of life,^8 9^ high levels of psychiatric and physical comorbidity for other psychiatric and physical disorders^10^ and increased mortality.^11 12^ Importantly, obesity and eating disorders frequently co-occur and share common risk factors, such as unhealthy weight control, body dissatisfaction and dieting practices,^13 14^ and emerging evidence points to genetic correlations between obesity and eating disorders such as binge eating disorder and bulimia nervosa.^15^ Therefore, public health efforts to reduce obesity and public health efforts to reduce eating disorders should not be seen as conflicting, but rather joint efforts to improve both physical and mental health in the population.^16^,^18^

However, to date, few policies targeting obesity have considered the potential impact on eating disorder psychopathology. One view is that the public health benefits of policies aiming to reduce obesity need to be balanced against potential harms. By way of illustration, the Royal Society for Public Health, an independent charity promoting public health in the UK, issued a statement in 2020 distancing themselves from their own position paper published in 2016 on the use of physical activity equivalent calorie labels due to the potential harm of such labels to people with eating disorders.^19^ In their revised statement, they highlight that while such labels may be effective in reducing obesity, the potential negative impacts on mental health would not be worth the tradeoff. However, there may possibly be a more complex picture, in which out-of-home nutrition labelling may have a balance of benefits and harms for individuals with eating disorders. At present, while there have been reviews on the effectiveness of out-of-home nutrition labels in terms of impact on obesity,^20 21^ only one review to date has considered the impact of such labels on people with eating disorders.^22^ However, the review was focused on alcoholic drink labels rather than out-of-home nutritional labelling policies more broadly. Hence, there is a need for a systematic review to provide a more comprehensive understanding of the potential public health impacts of such policies.

## Aim

The aim of the present review is to understand the impact of out-of-home nutrition labelling on individuals with eating disorders or disordered eating. Disordered eating refers to the symptoms of eating disorders whereby individuals may not meet full criteria for an eating disorder but show subclinical symptoms. It has been suggested that disordered eating exists on a continuum ranging from normative eating behaviour at one end and a diagnosed eating disorder at the other end of the spectrum.^23^ For the current review, we aimed to include individuals with a clinical eating disorder and those with disordered eating to fully understand the impacts of out-of-home nutritional labels. This knowledge will provide potentially important information to balance reviews that have previously reported on the impact of nutrition labels on obesity rates via purchasing behaviour.

## Methods

### Search strategy

The systematic review was conducted following a prepublished protocol (PROSPERO CRD42023389596). Results are reported according to Preferred Reporting Items for Systematic Reviews and Meta-Analyses (PRISMA) guidelines.^24^ Searches were conducted on 29 June 2023 in eight databases: MEDLINE, EMBASE, APA PsycINFO, Web of Science (Core Collection), ProQuest Dissertations and Theses Global, Scopus and CINAHL. Unpublished studies were searched for on Google Scholar and PsyArXiv. Searches across all databases were repeated on 11 October 2023. Records were deduplicated using Covidence software, before two authors (EW and KC) independently screened articles in two stages, first using titles and abstracts and then full-text records. Non-consensus was resolved by a third reviewer (TJ). Finally, we searched the reference lists of studies assessed during the full-text stage to identify additional studies.

### Inclusion/exclusion criteria

Studies were included if they reported qualitative or quantitative data on the impact of any form of out-of-home nutrition labelling of food or beverages on individuals with eating disorders, those experiencing disordered eating, or reported outcomes relating to eating disorders or disordered eating. In line with the English government definitions, the out-of-home sector was defined as follows: ‘generally considered to be any outlet where food or drink is prepared in a way that means it is ready for immediate consumption, on or off the premises’. Examples include restaurants and takeaway outlets. Nutrition labels could include calorie content, traffic-light ratings, equivalent exercise values or any other information designed to inform the consumer about the nutritional content of food or beverage items in the out-of-home sector. Studies investigating the impact of nutrition labelling on prepacked food and beverages were excluded.

### Data extraction

Data were extracted independently by two authors (EW and KC). For all included studies, we extracted the following information into a table: author, year of publication, country, sample size, sample characteristics (age, gender, ethnicity and socioeconomic status), measure of eating pathology or how eating disorder status was assessed, study design and method of data collection. For qualitative studies, we additionally extracted data pertaining to themes to allow for a metasynthesis.

### Quality assessment

The mixed-methods appraisal tool (MMAT) was used to appraise the methodological quality of included studies.^25^ The MMAT sets five quality appraisal questions for five study types: mixed methods, quantitative descriptive, quantitative non-randomised, quantitative randomised control trials and qualitative studies. All studies were assessed by the first author (NT) and at least one other author (FD, IP, HS, EM and TJ).

### Patient and public involvement

Individuals with lived experience of eating disorders were involved in the design, reporting and dissemination plans of this research.

## Results

**Figure 1:**
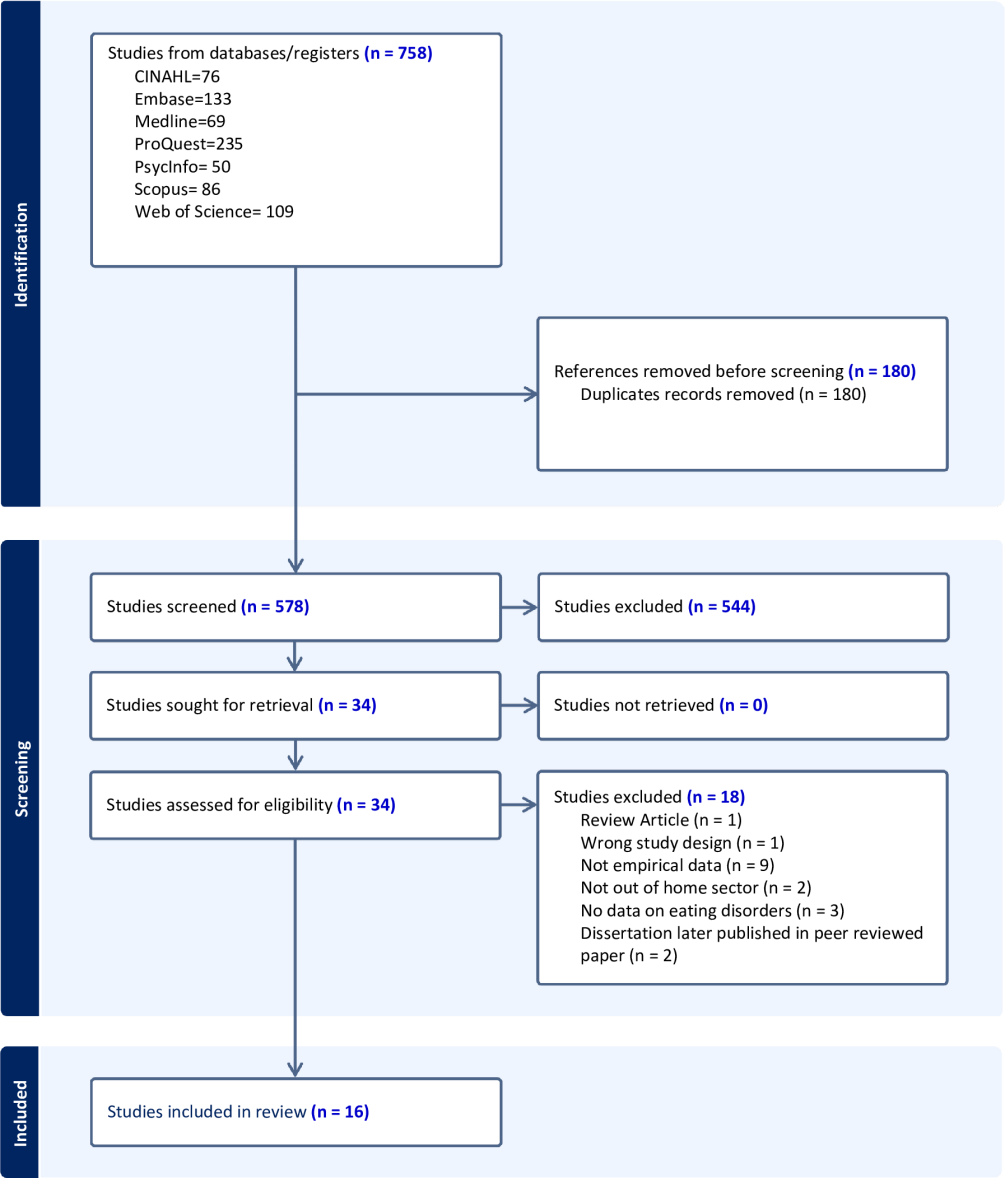
PRISMA flowchart. PRISMA, Preferred Reporting Items for Systematic Review and Meta-Analysis Protocols.

The initial search identified 758 studies, of which 578 remained after deduplication. Out of these, 34 studies were deemed relevant after screening titles and abstracts. After full-text review, 16 studies were included in the final review. See PRISMA diagram in figure 1 for further information. Details of the search strategy are presented in online supplemental file 1. Searches of Google Scholar and PsyArxiv in June 2023 identified three preprints. However, by October 2023, all three studies were published and picked up in the database searches. Details of studies excluded at full-text screening are available in online supplemental file 2.

### Study characteristics

Study characteristics are summarised in table 1. In total, there were five experimental/quasi-experimental studies, five cross-sectional studies and six qualitative/mixed-methods studies. Study samples varied in terms of age; however, all studies were conducted exclusively among adults. Most studies were conducted in Western countries (eight in the USA, one in Canada and five in the UK), with two studies conducted in Saudi Arabia.

**Table 1:** Study characteristics

First author, year	Country	Study design	N	Sex	Age, years Mean (SD)	Sample characteristics	ED measure
Al-Otaibi, 2021^30^	Saudi Arabia	Quasi-experiment	333	333 F0 M(100% F)	20 (NR)	Community sample of university studentsRace/ethnicity: NRSES: Monthly household income >5000 riyals (>70% of participants)	None
Bakhamis, 2022^33^	Saudi Arabia	Cross-sectional survey	379	190 F189 M(50.13% F)	22 (NR)	Community sample of university studentsRace/Ethnicity: NRSES: Most participants parents were university educated (43.5% of fathers and 44.6% of mothers)	EAT-IV
Duffy, 2023^39^	UK	IPA analysis	11	10 F1 M(90.91% F)	Recovered from ED: 31 (8.77)Currently has ED: 34, (6.42)	Community sampleIdentified as having current ED: AN (n=2), OSFED (n=2), atypical anorexia (n=1), ED (n=1) and recovered from ED: AN (n=4), ED (n=1)Race/ethnicity: NRSES: NR	Self-reported ED diagnosis
Frances, 2024^38^	UK	Qualitative survey	399	270 F15 NB5 NC5 M1 preferred not to state	16-24: 6125-34: 14235-44: 6245-54: 2255-64: 1165+: 1	Community sampleSelf reported diagnoses: AN (55%), BN (21%), BED (8%), OSFED (6%), ARFID (2%), RD (<1%), other (7%)Race/ethnicity: White English (n=195), White British (n=43), White background (n=34), White & Asian (n=5), Indian (n=3), Arab (n=2), mixed ethnic background (n=3)SES: NR	Self-reported ED diagnosis
Haynos, 2017^32^	USA	Experimental	716	716 F0 M(100% F)	21.52 (5.66)	ED sample: anorexia (n=7), BN (n=23), BEN (n=66)Race/ethnicity: White (n=455)SES: NR	EDE-Q
Larson, 2018^26^	USA	Cross-sectional survey	1830	1042 F788 M(56.92% F)	31.0 (1.6)	Project EAT, fourth waveCommunity sample of middle school studentsRace/ethnicity: White (n=1,241), Black/African American (n= 154), Asian (n=355.02), Asian American (n= 268), Mixed/other (n= 153)SES: household income: $50,0000-$99,999 (n=712) educational attainment: 4-year college degree (n=654)	EAT-IV
Lillico, 2015^27^	Canada	Pre-post intervention study	299	299 F0 M(100% F)	NR	Community sample of undergraduate university studentsRace/ethnicity: baseline (White (n=67), other (n=64)), follow-up (White (n=79), other (n=89))SES: NR	Eating attitudes test-26
Martinez, 2013^34^	USA	Cross-sectional survey	487	F/M (NR)	NR	Community sample of undergraduate university studentsRace/ethnicity: NRSES: NR	None
Moore, 2022^36^	USA	Cross-sectional survey with eye tracking	56	F/M (NR)(57% F)	NR	Community sample of undergraduate university studentsRace/ethnicity: NRSES: NR	Revised restraint scale
Polden, 2023^40^	UK	Sentiment analysis	NR (276 replies/quote‐tweet)	F/M (NR)	NR	Examined responses to social media posts on Twitter (tweets)Race/ethnicity: NRSES: NR	None
Putra, 2023^37^	UK	Mixed methods	1273	883 F349 M36 other(69.4% F)	34.59 (11.15)	Community sample from Prolific Academic and social media583 with ED diagnosis; AN (32%), BN (24%), and BEN (34%) were the most common diagnoses), general mental health symptoms for all; (anxiety disorder (76%), depression (79%) were most reportedRace/ethnicity: with ED: White (n=521), non-White (n=59), without ED: White (n=631), non-White (n=55)SES: education (with ED): below degree level (n=206), degree level (n=374); education (without ED): below degree level (n=286), degree level (n=400), household income (with ED): lowest (n=146), middle (n=152), highest (n=148); household income (without ED): lowest (n=198), middle (n=211), highest (n= 176)	EDE-Q-7
Raffoul, 2022^41^	USA	Mixed methods	13	10 F3 M(77% F)	18.8 (1.3)	Community sampleRace/ethnicity: South Asian (n=4), Caribbean (n=3), White (n=3), East Asian (n=2), Southeast Asian (n=1)SES: NR	Eating attitudes test-26 and BESAA score
Roberto, 2013^35^	USA	Cross-sectional survey	371	F/M (NR)(86% F)	33.2 (12.1)	Community sampleSelf reported: BED (n=52), BN (n=25), PD (n=17), No ED (n=277)Race/ethnicity: White (80%), Hispanic (5%), African American (5%), Asian (5%), Other (4%)SES: NR	EDE-Q
Robinson, 2022^31^	UK	Experimental study	1084	557 F527 M(51.38% F)	36 (14)	Community sample recruited through online platformDiagnosed with ED (n=23)Race/ethnicity: White (n=964), NR (n=120)SES: highest education (A level or higher) (n=652), household income (M: GBP £46 562, SD: GBP £80 767)	None
Seward, 2018^28^	USA	Mixed methods	Preintervention = 530; postintervention = 764	F/M (NR)(pre-intervention: 65% F; postintervention: 68% F)	20 (NR)	Community sample of university studentsRace/ethnicity: NRSES: NR	None
Shoychet, 2023^29^	USA	Experimental study	59	59 F0 M(100% F)	NRRange: 17-21	Community sample of undergraduate psychology studentsRace/ethnicity: White (n=22), South Asian (n=5), Chinese (n=15), Black (n=3), Middle Eastern (n=4), Southeast Asian (n=4), Korean (n=3), multiple (n=3)SES: NR	ESPI

AN, anorexia nervosa; ARFID, avoidant/restrictive food intake disorder; BEN, binge eating disorder; BESAA, Body-Esteem Scale for Adolescents and Adults; BN, bulimia nervosa; ED, eating disorder; EDE-Q, Eating Disorder Questionnaire; EDE-Q-7, Eating Disorder Questionnaire - 7 item version; ESPI, Eating Pathology Symptoms Inventory; F, female; IPA, interpretative phenomenological analysis; M, male; NB, non-binary; NC, non-conforming; NR, not reported; OSFED, other specified feeding or eating disorder; PD, purging disorder; RD, rumination disorder; SES, socioeconomic status.

### Quality assessment

The quality assessment ratings of the included studies are presented in table 2. Studies were generally rated as moderate to high quality, with some methodological flaws evident across studies. For example, quantitative studies and quantitative aspects of mixed-method studies lacked representative samples, with one exception.^26^ Further, studies generally did not report on missingness of data. No studies were excluded from the review due to study quality.

**Table 2:** Quality assessment ratings of included studies by study type

	Qualitative					Quantitative, randomised					Quantitative, non-randomised					Mixed methods				
First author, year	1	2	3	4	5	1	2	3	4	5	1	2	3	4	5	1	2	3	4	5
Al-Otaibi, 2021^30^											No	No	CT	No	Yes					
Bakhamis, 2022^33^											No	Yes	CT	No	Yes					
Duffy, 2023^39^	Yes	Yes	Yes	Yes	Yes															
Frances, 2024^38^	Yes	Yes	Yes	Yes	Yes															
Haynos, 2017^32^						CT	CT	CT	Yes	Yes										
Larson, 2018^26^											Yes	Yes	CT	Yes	Yes					
Lillico, 2015^27^											No	Yes	CT	Yes	Yes					
Martinez, 2012^34^											No	Yes	CT	No	Yes					
Moore, 2022^36^											No	Yes	CT	Yes	Yes					
Polden, 2023^40^																CT	Yes	Yes	Yes	No
Putra, 2023^37^																CT	Yes	Yes	Yes	No
Raffoul, 2022^41^																Yes	Yes	Yes	Yes	No
Roberto, 2013^35^											No	No	CT	Yes	Yes					
Robinson, 2022^31^						Yes	Yes	Yes	CT	Yes										
Seward, 2018^28^																CT	Yes	Yes	Yes	No
Shoychet, 2023^29^						Yes	Yes	Yes	No	Yes										

Qualitative: 1 = appropriate design, 2 = adequate data collection. 3 = findings derived from data, 4 = appropriate interpretation, 5 = coherence.

Quantitative – randomized: 1 = appropriate randomization, 2 = comparable groups, 3 = complete data, 4 = blinding, 5 = adherence.

Quantitative – non-randomized: 1 = representative sample, 2 = valid measures, 3 = complete data, 4 = confounders, 5 = exposure occurred.

Mixed methods: 1 = adequate rationale, 2 = integrated research question, 3 = adequate interpretation, 4 = inconsistencies addressed, 5 = quality criteria met for qualitative and quantitative component

CT, cannot tell.

### Experimental studies

In total, five studies employed an experimental or quasi-experimental design whereby participants were presented with calorie-labelled menus to measure differences between groups (ie, no calorie-labelled menu vs calorie-labelled menu) or pre–post differences. In addition, one of the three mixed-methods studies also included a pre–post design, which is reported below. Using a pre–post design in a university food hall where calorie labels were introduced for 1 week, Lillico *et al*^27^ found no differences in calorie consumption, body image satisfaction, anxiety, positive effect or unhealthy weight-related behaviours among undergraduate students (n=299). Similarly, Seward *et al*^28^ used a mixed-methods study to assess the introduction of nutritional labels in university cafeterias. Specifically, they assessed students in two groups before and after the introduction of the nutrition labels depending on whether they had cafeterias introduce traffic-light labels (ie, intervention group) or not (ie, control group). Most participants were in favour of traffic-light labels, with 50% of students (n=229) in the intervention group in favour of continuing the policy. However, many people raised concerns about those with eating disorders. 16% reported concerns that traffic-light labels put people at risk for developing eating disorders, 47% reported concerns that traffic-light labels exacerbate existing eating disorders and 35% reported concerns that traffic-light labels would make recovery from an eating disorder more difficult.

Using an experimental design, Shoychet *et al*^29^ randomly allocated female undergraduate students (n=59) to a calorie-label or no-calorie-label condition in which they were presented a food menu with or without calories and selected preferred foods. No differences were observed in the two groups regarding food choice, body dissatisfaction or disordered eating. Similarly, Al-Otaibi *et al*^30^ used a quasi-experimental design among female university students whereby those with high-weight concerns (ie, heightened worry about one’s weight) were presented a menu with calorie labels and those with low-weight concerns a menu without calorie labels. Given the difference in the two groups, it is unclear whether any differences observed are due to the calorie labels or weight concerns of the participants. However, the study also investigated general behavioural responses to calorie labels in both conditions. In both groups, participants reported changes in behaviour when using calorie labels at restaurants. These included avoiding high-calorie options (low-weight concern group: 6.5% and high-weight concern group: 2.8%), not ordering high-calorie foods (low-weight concern group: 24.2% and high-weight concern group: 19%) and deciding on smaller portion sizes (low-weight concern group: 29% and high-weight concern group: 60.2%).

Using a slightly different approach, Robinson *et al*^31^ compared different label conditions (control vs kcal labelling vs kcal+physical activity equivalency labelling) for alcoholic drinks among adults. However, no comparisons were made between the groups in terms of disordered eating. Instead, the overall sample reported their expected behavioural response to calorie labels on alcoholic drinks. In total, 13% of participants reported that they would skip meals on drinking days and 2% would engage in purging behaviours to control their weight.

Only one study examined the impact of nutrition labels on people with eating disorders directly. Haynos and Roberto^32^ surveyed adults (n=716) who either met or did not meet criteria for an eating disorder and assessed responses to calorie labels through a virtual menu. Participants who met criteria for anorexia nervosa or bulimia nervosa ordered significantly fewer calories when presented with calorie labels compared with menus presented without calorie labels. In contrast, participants who met criteria for binge eating disorder ordered significantly more calories when presented with calorie labels compared with menus presented without calorie labels. No differences were observed for participants not meeting criteria for an eating disorder.

Overall, experimental studies showed no differences in eating pathology between individuals exposed to calorie labels compared with those not exposed to calorie labels. However, in a study among individuals with eating disorders, Haynos and Roberto^32^ found significant differences in behaviours between conditions, whereby individuals with restrictive eating pathology chose lower calorie options and individuals with binge eating pathology choose higher calorie options in the labelled condition.

### Cross-sectional survey studies

In total, five studies employed a cross-sectional survey design where participants were asked to self-report on their behaviours in response to nutrition labels. In addition, one of the three mixed-methods studies also included a cross-sectional survey, which is reported below.

Bakhamis *et al*^33^ surveyed university students (n=379) in Saudi Arabia regarding the use of calorie-labelled menus and disordered eating. Most participants (74%) had noticed calorie labels on menus within the previous month and 45.7% of participants reported using calorie labels to avoid high-calorie options. Both greater awareness and use of labels to avoid high-calorie options were associated with higher weight concerns and disordered eating. Similar results were found by Larson *et al*^26^ who used data from Project EAT, a longitudinal study examining eating disorder risk factors in a large US community sample (n=1830). Participants were asked whether they noticed calorie information in restaurants and the impact of this information, with results weighted for non-response. Around half of the participants (52.7%) reported noticing calorie information at a restaurant within the previous month. Among those, 50.1% reported that they used the information to avoid high-calorie options, equivalent to 26.4% of the total sample.

Four studies examined the impact of calorie labels on individuals with eating disorders specifically. Martinez *et al*^34^ surveyed undergraduate students at a US university where the university food halls provide nutritional labels. Eating disorder status was not assessed within this sample. While almost all participants were in favour of the labels, 29% felt that labels could exacerbate existing eating disorders and 34% believed that the labels would make recovery more difficult. In contrast, Roberto *et al*^35^ found no difference between adults with or without an eating disorder in terms of support for calorie labels, which were supported by almost all participants. Using eye-tracking information on calorie menus, Moore *et al*^36^ found that individuals who engage in restrictive eating spend more time looking at calorie labels on menus compared with individuals who do not engage in restrictive eating. Additionally, individuals who engage in restrictive eating selected lower calorie options compared with those not engaging in restrictive eating. In the UK, Putra *et al*^37^ conducted a mixed-methods study with a cross-sectional survey, which found that individuals with an eating disorder were less likely to agree with the calorie labelling policy compared with individuals with other mental health concerns. Over half of the participants with an eating disorder reported that calorie labels may worsen their symptoms.

Overall, results from the cross-sectional studies showed that noticing and using calorie labels on menus was related to eating pathology, whereby individuals who had higher levels of disordered eating or body image concerns were more likely to both notice calorie information and change their behaviour due to calorie labels, mostly to avoid high-calorie food and choose low-calorie options instead.

### Qualitative/mixed-methods studies

Six studies with qualitative data were identified during screening (see table 1), undertaken in the UK^37^,^40^ and the USA.^28 41^ Two of the studies investigated the impact of nutrition labelling interventions,^28 41^ whereas the others captured experiences regarding the introduction of calories on menus legislation. Thematic synthesis was conducted by three authors (FD, EM and IP) to synthesise the identified qualitative studies.^42^ The extracted data were read multiple times and coded line by line. The descriptive themes were developed from the initial codes. Analytical themes were generated to ‘go beyond’ the extracted data and identify overarching themes. In total, five themes were identified. These included *being drawn to calories*, *facilitating the eating disorder*, *reassurance*, *social eating* and *frustration*.

#### Theme 1: being drawn to calories

Many participants were already highly attuned to calorie content as a part of their eating disorder. The prominent placement of calorie labelling on menus meant that participants felt drawn to be *hyperaware of calories,* which became the dominant factor driving their food choice, a *Blinkered choice*.

##### Hyperaware of calories

Participants with eating disorder experience were immediately drawn to the calorie information. They felt unable to ignore it^37 39 41^ and perceived it to be ‘*in your face’* with a ‘*spotlight’* on it.^39^ Some participants experienced guilt and anxiety after viewing labels; ‘*I become hyperaware of the idea of the calories, I imagine my body ballooning up, I feel dirty’*.^37^ To overcome the distraction, one participant described taking a pen to mark out the numbers.^39^ Calorie information was felt to be unavoidable and drew attention away from other aspects of their eating out experience.

##### Blinkered choice

Participants in most of the studies noted the impact of nutrition labelling on their food choices.^2837^,^39 41^ They reflected that their selection would be dictated by calorie content, not preference.^38 39^ Participants described ignoring their bodily signals and instead choosing the lowest calorie option, resulting in them feeling hungry.^38^ A participant in Seward’s study^28^ of traffic-light labels reflected on a friend who *‘completely stopped eating anything unless it was green … to the point where we were concerned about her because she would not take anything that was red or yellow’*. Nutrition labelling allowed the eating disorder to dictate menu choices in favour of the lowest calorie option removing any sense of ‘*food freedom’*.^38^ Some participants struggled to eat anything at all, if items were perceived as too high in calories.^37^

### Theme 2: facilitating the eating disorder

Across the studies, participants were concerned that nutrition labelling would lead to either the development or relapse of an eating disorder. This was partly owing to the perception that the policy was *reinforcing eating disorder beliefs* and that nutrition labelling was used as an *enabler of the eating disorder*.

#### Reinforcing eating disorder beliefs

In studies that explored the implementation of nutrition labelling in England, participants held the perception that the legislation reinforced eating disorder beliefs.^38 39^ Many participants were frustrated with the government and public health in encouraging a problematic culture of eating. Participants interpreted that the English calorie labelling policy implies that low-calorie food choices and calorie counting were the only way to remain healthy, echoing their eating disorder cognitions.

It just reinforces the thoughts that I have, like, I don't know, I don't need that because this is what it says here.^39^

In addition, participants were confused by having their eating disorder thoughts challenged in treatment and then supported in public health legislation. One participant explained in terms of calorie counting ‘*I find it really confusing because in my mind I'm like I've had so much intensive treatment and I'm having all these people tell me that that is not the right thing for me to do’*.^39^ At best, participants found the inconsistency in advice confusing. At worst, the legislation was perceived to justify their eating disorder thoughts.

#### Enabler of the eating disorder

Participants reported that the displaying of nutrition labelling enabled eating disorder behaviours.^2837^,^39 41^ Nutrition labelling acted as a prompt for their eating disorder, making their thoughts ‘*louder and louder’*,^39^ generating anxiety or leading to engagement in disordered behaviours as ‘…*seeing the (calorie) info has often triggered days-long spirals’*.^38^ The nutrition labelling was a reminder of what they had consumed or gave them the information they needed to calorie count.

‘I am on a binge restrict cycle with my eating disorder. Calorie information feeds into this as it facilitates restriction, but also if I have a calorie expensive meal then it will trigger a bingeing episode as the damage is already done’.^37^

Though some participants felt their behaviours were a direct consequence of nutrition labels,^41^ others were more ambivalent.^39^ There was an awareness across the studies that the nutrition labelling was able to provide information which an eating disorder was then able to use to engage in disordered behaviour.

Those who were in recovery described increased mental effort when they experienced nutrition labelling and felt it had challenged their progress and efforts, with one participant describing their recovery as having been ‘*attacked’* by the presence of calories on menus.^39^ For other participants, they had to remove themselves from eating out, reporting that ‘*It’s definitely set my recovery back by a long way and I only feel safe eating at home now’*.^38^

### Theme 3: reassurance

There were also instances in which nutrition labelling was perceived as beneficial and provided reassurance.^37 38^ Some participants reported that the presence of calories on menus left them feeling *safer*, enabling a *freedom* to engage socially with others and begin to take perceived ‘risks’ around food choices.

#### Perceived safety in knowledge

Some participants with bulimic/binge eating pathology described nutrition labelling as providing a sense of safety with one individual reporting it prevented them ‘*going overboard in the moment’*.^37^ Another participant noted;

‘Most of my anxiety from eating out comes from not knowing what is in the food… This then often leads to making poor decisions around food… and/or overestimating the amount of calories in a meal, which then means restricting more during other meals. I would much rather be empowered to make conscious decisions about my food intake’.^37^

#### Freedom

The reassurance of calorie information facilitated some participants to eat socially with friends, enabling them to take part in activities they may not have been able to do otherwise.^38^ ‘It *allows me to go out and eat rather than having to meticulously track my calories at home and guess outside’*.^38^ It empowered some individuals to be exposed to and acknowledge calories, rather than avoid them. In addition, participants reported being able to take risks and try ‘*unsafe’ foods healthily by including them in their calorie count for the day*’.^37^

### Theme 4: social eating

Participants felt that nutrition labelling served to *spoil the enjoyment* of eating out with others. Nutrition labelling was noted to *facilitate diet talk* among their companions*,* and some individuals feared that requesting calorie free menus might lead to their eating disorder being *exposed*. For some participants, they avoided eating out altogether resulting in a sense of *isolation*.

#### Spoilt enjoyment

Participants in half the studies reported that nutrition labelling had undermined their enjoyment of eating out^37^,^39^; ‘*Eating out is a fun social experience. It is not something I do every day and I do not want this spoiling for me by having to worry about calories’*.^37^ A participant reflected that ‘*Going out and eating was a big part of my initial recovery: learning to not have to pre-look at a menu, being more spontaneous and enjoying socialising*’.^38^ With nutrition labelling, they were cautious not to ‘*alienate’* themselves ‘*by getting anxious about ordering and cause tension’* with the people they were with.^38^ Their preoccupation with how they might be perceived hampered their enjoyment of eating out with others.

#### Facilitating diet talk

A few studies reported that nutrition labelling was perceived as having increased diet talk by companions.^38 39^ Diet talk was described as difficult and left some feeling ‘*unable to join in the conversation or sometimes feeling highly distressed*’.^38^ In Roberto *et al*’s study,^35^ a participant recalled feeling uncomfortable ‘*having to ask other people at the table to stop discussing the calories which makes the rest of the meal very awkward’,* concluding that they felt *‘people would rather just stop inviting me to stuff’*.

#### Exposed

Participants were concerned that their reactions to the nutrition labelling, particularly when asking for a calorie-free menu, would expose their eating disorder.^38 39^ They were worried that they would ‘*draw attention’*^38^ when asking for a calorie-free menu, as they viewed it as ‘*exposing’*^39^ and felt a sense of ‘*shame and embarrassment’*.^38^

‘I personally don't like to draw attention to the ED around friends. So asking for a no calorie on menu alternative feels like I'm shouting ‘I have an ED’ and what if they say no … that they don't have one? Then my face and body language would give it away!’^36^

#### Isolation

As a result of nutrition labelling, participants reported isolating themselves and avoiding eating out, or being uninvited to social events which involved eating out.^38^ As one participant explained; ‘*One thing the ED loves is isolation. And eating out is a more popular thing to do with friends at my age … I now don't go out’*.^38^ Participants felt that eating out was an important part of their recovery and a key social activity. By not engaging in it, they felt isolated and concerned that it would make their eating disorder ‘*even worse through the loneliness’*.^38^

### Theme 5: frustration

In studies that explored the impact of the implementation of calorie labelling regulations in England, participants described frustration that this had been forced on them due to their perception that it was both *ineffective* and *lacked consideration for those with eating disorders*.

#### Perceived ineffectiveness

Participants’ frustration at the implementation of the legislation appeared to be partly driven by the perception that the policy was misguided in addressing the underpinning causes of ‘*the obesity epidemic’* and ineffective for its target population.^38^,^40^

‘Doing this in the name of tackling ‘the obesity epidemic’ shows a wilful ignorance on behalf of the government … I worry that this will have the worst impact on fat people, who already suffer disproportionately from eating disorders and public shaming’.^38^

#### Lack of consideration for those with eating disorders

There was also the sense that the legislation lacked consideration for the impact it would have on individuals with eating disorders. It was felt that the ‘*eating disorder specialist should have been listened to’*^40^ and participants were frustrated that the government was willing to risk detrimental impact on people with eating disorders; *‘it is kind of detrimental to people affected by EDs, then what’s the point?’*^39^ Participants reflected that they were angered that the impact on them was not being considered as important enough.

## Discussion

This review summarised the current evidence of a variety of out-of-home nutrition labels on people with eating disorders. Overall, the findings highlight the limited evidence available. We identified 16 studies in total, most of which focused primarily on the effectiveness of the labels in reducing caloric intake and few of which recruited individuals with lived experience of eating disorders specifically. Most experimental studies showed no differences between menus with or without calories on disordered eating or body image concerns. However, individuals with current eating pathology changed their behaviours if presented menus with calorie labels. Thus, behavioural changes and impacts on eating pathology may not be observed in the general population and appear to be specific to those with eating disorders.

Findings from cross-sectional studies support this, whereby eating pathology was positively associated with noticing calorie labels and changing behaviour to select lower calorie options. However, few studies focused specifically on individuals with eating disorders and no broader impacts on mental health were examined. Future research is needed to understand whether calorie labels are viewed positively or negatively, and whether they could potentially reinforce or perpetuate eating disorder psychopathology.

More nuanced information on the impacts of nutrition labels was reported by the reviewed qualitative and mixed-methods research. Across studies, individuals with eating disorders reported several negative impacts of nutrition labels, such as avoiding restaurants and labels triggering their eating disorder cognitions. These findings were consistent across people with current and past experiences of eating disorders. Additionally, many people in the general population voiced concerns for those with eating disorders regarding calorie labels. There is emerging evidence that warrants concern regarding the impact out-of-home nutrition labels have on individuals with eating disorders. People with eating disorders also voiced their frustration with not being listened to, feeling that concerns about eating disorders were perceived as less important in the light of obesity prevention.

However, existing evidence also points towards potential heterogeneity in reported impacts. Studies by Putra *et al*^37^ and Haynos and Roberto^32^ suggest that there may be differences in people who engage in binge eating and those who engage in restrictive eating. Further, some people with eating disorders have reported positive impacts of calorie labels whereby labels were reassuring and may enable social activities that would not be possible otherwise. More research is needed to understand the heterogeneity in the impacts of nutrition labels and determine whether certain groups of people are particularly at risk and conversely, whether there are groups of people benefitting from such labels. Understanding the benefits of labels is made harder by the possibility that some perceived benefits, such as calorie counting reducing guilt in the short term, may also be linked to longer term risks, such as dietary restriction.^35^ Qualitative research methods may be best suited to exploring this complexity with participants.

Several included studies also point towards potential explanations or mechanisms explaining the negative impacts of out-of-home nutrition labels. For example, the metasynthesis points towards calorie labels reinforcing existing eating disorder beliefs. Similarly, both the metasynthesis and experimental research from Moore *et al*^36^ highlight that individuals with eating disorders are more drawn to viewing calories or traffic-light symbols and may pay more attention to them. Hence, the oversimplified nature of out-of-home nutritional labels (as opposed to nutritional labels of packaged foods) may be particularly problematic.

While the current review had many strengths, such as the inclusive search strategy and focus on different study designs, some limitations should be considered. First, most included studies focused on local or country-specific calorie labelling policies. Cultural and societal context should be considered when interpreting the findings. Second, most studies relied on convenience samples of undergraduate students, which are unlikely to represent the diverse experiences of people across the socioeconomic and age spectrum. Third, while the current review included studies with different types of nutrition labels (eg, traffic-light system and numeric labels), we were unable to compare the impacts of different labelling policies. Instead, conclusions and recommendations are applied to nutrition labels overall, without specific guidance on certain labelling policies.

In terms of future directions for research, the current review highlights several areas to explore. First, none of the reviewed studies included children or adolescents. Eating disorders typically develop during adolescence, with a peak age of onset at 15.5 years.^43^ Children and adolescents may be impacted the most by calorie labelling policies, as they tend to be more vulnerable to societal messaging and stigma.^44^ Future research should examine how children and adolescents with eating disorders are impacted by calorie labelling policies and whether this differs from adults. Second, more research among individuals with eating disorders needs to be conducted. As discussed above, heterogeneity of eating disorder presentations is yet to be accounted for and unlikely to occur in convenience samples. Third, the quality assessment of the reviewed studies pointed towards important gaps in the current research. For example, representative samples and inclusion of more diverse experiences were lacking. This will be an important challenge for future research, as information regarding the impact of public health policies should ensure that diverse experiences are included, and decisions are not solely based on ‘mainstream’ voices. Eating disorder research and advocacy is primarily dominated by young women with restrictive eating disorders.^45 46^ This is also reflected in the qualitative studies with individuals with eating disorders in the current review, which were dominated by women with anorexia nervosa. Despite this prevailing stereotype of eating disorder experiences, it is well documented that binge eating disorder is more prevalent compared with anorexia nervosa in community samples,^10 47^ and that up to a quarter of those with eating disorders are men.^48^ It will be critical for future research to ensure that diverse experiences are captured to establish the impact of nutrition labels on people with eating disorders more accurately, not just women with restrictive eating disorders. Third, most studies did not consider the nutrition label knowledge and literacy of the participants in the studies, which may impact their choices. Indeed, individuals with eating disorders may have higher levels of nutrition literacy compared with those without eating disorders,^49^ which may impact their perception of nutrition labels.

The current review has important implications for public health policies on nutrition labels. The main aim of this review was to investigate the impacts of out-of-home nutrition labelling policies on individuals with eating disorders. Overall, the evidence we have reviewed points towards predominantly negative impacts of such labels on people with eating disorders, although some positive impacts have been reported. Policymakers should use this information alongside previous reviews that have examined the effectiveness of such policies in targeting obesity rates^20 21^ to determine whether nutrition labelling policies strike the balance between potential benefits and harms. The current review does not, however, offer potential solutions to currently implemented out-of-home nutritional label policies that would reduce the negative impacts on people with eating disorders. Alternative policies that have been suggested include having calories available on request^50^ or accessible via a QR code or online.^38^ Further research and consultation with individuals with lived experience of obesity and eating disorders are required to identify policies that benefit the general population without causing harm to those with eating disorders.

## Supplementary material

10.1136/bmjph-2023-000862 online supplemental file 1

10.1136/bmjph-2023-000862 online supplemental file 2

## Data Availability

No data are available.
