# Supplementary material for: Impact of out-of-home nutrition labelling on people with eating disorders: a systematic review and meta-synthesis
Source: BMJ Public Health. 2025 Jan 29;3(1):e000862. doi: 10.1136/bmjph-2023-000862 (PMC11816730; doi:10.1136/bmjph-2023-000862)
Supplement: online supplemental file 1 [file bmjph-3-1-s001.pdf]

Supplementary 1 – Search strategies

PsycINFO

#	Query
1	exp Eating Disorders/
2	anorexi*.mp.
3	bulimi*.mp.
4	binge eat*.mp.
5	eating disorder*.mp.
6	exp Feeding Disorders/
7	exp Anorexia Nervosa/
8	exp Bulimia/
9	exp Binge Eating/
10	avoidant restrictive food intake disorder.mp.
11	ARFID.mp.
12	OSFED.mp.
13	(other specified feeding or eating disorder*).mp.
14	pica.mp.
15	rumination disorder*.mp.
16	(unspecified feeding or eating disorder*).mp.
17	purging disorder*.mp.
18	night eating syndrome*.mp.
19	EDNOS.mp.
20	(eating disorder not otherwise specified).mp.
21	1 or 2 or 3 or 4 or 5 or 6 or 7 or 8 or 9 or 10 or 11 or 12 or 13 or 14 or 15 or 16 or 17 or 18 or 19 or 20
22	(calorie* adj2 label*).mp.
23	(nutrition* adj2 label*).mp.

24	(menu* adj2 label*).mp. [mp=title, abstract, heading word, table of contents, key concepts, original title, tests & measures, mesh word]
25	(traffic adj2 label*).mp. [mp=title, abstract, heading word, table of contents, key concepts, original title, tests & measures, mesh word]
26	(calorie* adj2 info*).mp. [mp=title, abstract, heading word, table of contents, key concepts, original title, tests & measures, mesh word]
27	(nutrition* adj2 info*).mp. [mp=title, abstract, heading word, table of contents, key concepts, original title, tests & measures, mesh word]
28	(calorie* adj2 menu*).mp.
29	22 or 23 or 24 or 25 or 26 or 27 or 28
30	21 and 29

Embase 1974 to 2023

#	Query
1	exp eating disorder/
2	anorexi*.mp.
3	bulimi*.mp. [mp=title, abstract, heading word, drug trade name, original title, device manufacturer, drug manufacturer, device trade name, keyword heading word, floating subheading word, candidate term word]
4	binge eat*.mp.
5	eating disorder*.mp.
6	exp feeding disorder/
7	exp anorexia nervosa/
8	exp bulimia/
9	exp binge eating disorder/
10	exp avoidant restrictive food intake disorder/
11	ARFID.mp.
12	OSFED.mp.
13	(other specified feeding or eating disorder*).mp.
14	pica.mp.

15	rumination disorder*.mp.
16	(unspecified feeding or eating disorder*).mp.
17	purging disorder*.mp.
18	night eating syndrome*.mp.
19	EDNOS.mp.
20	(eating disorder not otherwise specified).mp.
21	1 or 2 or 3 or 4 or 5 or 6 or 7 or 8 or 9 or 10 or 11 or 12 or 13 or 14 or 15 or 16 or 17 or 18 or 19 or 20
22	(calorie* adj2 label*).mp.
23	(nutrition* adj2 label*).mp.
24	(menu* adj2 label*).mp.
25	(traffic adj2 label*).mp.
26	(calorie* adj2 info*).mp.
27	(nutrition* adj2 info*).mp.
28	(calorie* adj2 menu*).mp.
29	22 or 23 or 24 or 25 or 26 or 27 or 28
30	21 and 29

Ovid MEDLINE(R) ALL 1946 to2023

#	Query
1	exp "Feeding and Eating Disorders"/
2	anorexi*.mp.
3	bulimi*.mp.
4	binge eat*.mp.
5	eating disorder*.mp.
6	exp Anorexia Nervosa/
7	exp Bulimia Nervosa/ or exp Bulimia/

8	exp Binge-Eating Disorder/
9	exp "Feeding and Eating Disorders of Childhood"/ or exp "Feeding and Eating Disorders"/ or exp Avoidant Restrictive Food Intake Disorder/
10	ARFID.mp.
11	OSFED.mp.
12	(other specified feeding or eating disorder*).mp.
13	pica.mp. or exp Pica/
14	rumination disorder*.mp.
15	(unspecified feeding or eating disorder*).mp.
16	purging disorder*.mp.
17	night eating syndrome*.mp.
18	EDNOS.mp.
19	(eating disorder not otherwise specified).mp.
20	1 or 2 or 3 or 4 or 5 or 6 or 7 or 8 or 9 or 10 or 11 or 12 or 13 or 14 or 15 or 16 or 17 or 18 or 19
21	(calorie* adj2 label*).mp.
22	(nutrition* adj2 label*).mp.
23	(menu* adj2 label*).mp.
24	(traffic adj2 label*).mp.
25	(calorie* adj2 info*).mp.
26	(nutrition* adj2 info*).mp.
27	(calorie* adj2 menu*).mp.
28	21 or 22 or 23 or 24 or 25 or 26 or 27
29	20 and 28

SCOPUS

(ALL (anorexi* OR "eating disorder*" OR bulimi* OR "binge eat*" OR "feeding disorder*" OR "anorexia nervosa" OR bulimia OR "binge eating disorder*" OR "avoidant restrictive food intake disorder*" OR arfid OR "Avoidant Restrictive Food Intake Disorder*" OR osfed OR "other specified feeding or eating disorder*" OR pica OR "rumination disorder*" OR "unspecified feeding or eating disorder*" OR "purging disorder*" OR "night eating syndrome*" OR ednos OR "eating disorder not otherwise specified") AND ALL (calorie* W/2 label* OR nutrition* W/2 label* OR menu* W/2 label* OR traffic W/2 label* OR calorie* W/2 info* OR nutrition* W/2 info* OR calorie* W/2 menu*))

Web of Science

anorexi* OR "eating disorder*" OR bulimi* OR "binge eat*" OR "feeding disorder*" OR "anorexia nervosa" OR bulimia OR "binge eating disorder*" OR "avoidant restrictive food intake disorder*" OR ARFID OR "Avoidant Restrictive Food Intake Disorder*" OR OSFED OR "other specified feeding or eating disorder*" OR pica OR "rumination disorder*" OR "unspecified feeding or eating disorder*" OR "purging disorder*" OR "night eating syndrome*" OR EDNOS OR "eating disorder not otherwise specified"

AND
calorie* NEAR/2 label* OR nutrition* NEAR/2 label* OR menu* NEAR/2 label* OR traffic* NEAR/2 label* OR calorie* NEAR/2 info* OR nutrition* NEAR/2 info* OR calorie* NEAR/2 menu*

CINAHL

S7	S3 AND S6
S6	S4 OR S5
S5	(MH "Product Labeling+") OR (MH "Food Labeling")
S4	calorie* N2 label* OR nutrition* N2 label* OR menu* N2 label* OR traffic N2 label* OR calorie* N2 info* OR nutrition* N2 info* OR calorie* N2 menu*
S3	S1 OR S2
S2	anorexi* OR "eating disorder*" OR bulimi* OR "binge eat*" OR "feeding disorder*" OR "anorexia nervosa" OR bulimia OR "binge eating disorder*" OR "avoidant restrictive food intake disorder*" OR ARFID OR "Avoidant Restrictive Food Intake Disorder*" OR OSFED OR "other specified feeding or eating disorder*" OR pica OR "rumination disorder*" OR "unspecified feeding or eating disorder*" OR "purging disorder*" OR "night eating syndrome*" OR EDNOS OR "eating disorder not otherwise specified"
S1	(MH "Eating Disorders+") OR (MH "Feeding and Eating Disorders of Childhood+") OR (MH "Avoidant Restrictive

	Food Intake Disorder") OR (MH "Binge Eating Disorder") OR (MH "Bulimia Nervosa")

Proquest Dissertation and Theses Global – Total hits: 225 (235 in October)

(title abstract(anorexi*) OR title abstract(bulimi*) OR title abstract(binge eat*) OR title abstract(eating disorder*) OR title abstract(ARFID) OR title abstract(Avoidant Restrictive Food Intake Disorder) OR title abstract(avoidant/restrictive food intake disorder) OR title abstract(eating disorder otherwise specified) OR title abstract(OSFED) OR title abstract(eating disorders not otherwise specified) OR title abstract(EDNOS) OR title abstract(pica) OR title abstract(night eating syndrome*) OR title abstract (rumination disorder*)) AND (calorie* NEAR/2 label* OR nutrition* NEAR/2 label* OR menu* NEAR/2 label* OR traffic* NEAR/2 label* OR calorie* NEAR/2 info* OR nutrition* NEAR/2 info* OR calorie* NEAR/2 menu*)
