# Supplementary material for: Impact of out-of-home nutrition labelling on people with eating disorders: a systematic review and meta-synthesis
Source: BMJ Public Health. 2025 Jan 29;3(1):e000862. doi: 10.1136/bmjph-2023-000862 (PMC11816730; doi:10.1136/bmjph-2023-000862)
Supplement: online supplemental file 2 [file bmjph-3-1-s002.pdf]

## Supplementary 2

## References of Included Studies


Al-Otaibi, H., Al-Sandal, T., & Elkatr, H. O. (2021). Is calorie labeling on menus related to weight disturbances among females in Saudi Arabia?. *Journal of Nutrition and Metabolism*, *2021*.

Bakhamis, T. M., Alqethami, A. M., Abd El-Fatah, N. K. (2022). Calorie Labeling on Restaurants Menu: Notice and Use among King Abdul-Aziz University Students in Jeddah City, Saudi Arabia. *Journal of Research in Medical and Dental Science.*

Duffy, F., Peebles, I., Maloney, E., Robertson, M. D., & Sharpe, H. (2023). Individuals with restrictive eating disorders' experience of the introduction of calories on menus in England: An interpretative phenomenological analysis study. *European Eating Disorders Review*.

Frances, T., O'Neill, K., & Newman, K. (2023). ‘An extra fight I didn't ask for’: A qualitative survey exploring the impact of calories on menus for people with experience of eating disorders. *British Journal of Health Psychology*.

Haynos, A. F., & Roberto, C. A. (2017). The effects of restaurant menu calorie labeling on hypothetical meal choices of females with disordered eating. *International Journal of Eating Disorders*, *50*(3), 275-283.

Larson, N., Haynos, A. F., Roberto, C. A., Loth, K. A., & Neumark-Sztainer, D. (2018). Calorie labels on the restaurant menu: is the use of weight-control behaviors related to ordering decisions?. *Journal of the Academy of Nutrition and Dietetics*, *118*(3), 399-408.

Lillico, H. G., Hanning, R., Findlay, S., & Hammond, D. (2015). The effects of calorie labels on those at high-risk of eating pathologies: a pre-post intervention study in a University cafeteria. *Public health*, *129*(6), 732-739.

Martinez, O. D., Roberto, C. A., Kim, J. H., Schwartz, M. B., & Brownell, K. D. (2013). A survey of undergraduate student perceptions and use of nutrition information labels in a university dining hall. *Health Education Journal*, *72*(3), 319-325.

Moore, K., Walker, D., & Laczniak, R. (2022). Attention mediates restrained eaters’ food consumption intentions. *Food Quality and Preference*, *96*, 104382.

Polden, M., Robinson, E., & Jones, A. (2023). Assessing public perception and awareness of UK mandatory calorie labelling in the out-of-home sector: Using Twitter and Google trends data. *Obesity Science & Practice*.

Putra, I. G. N. E., Polden, M., Wareing, L., & Robinson, E. (2023). Acceptability and perceived harm of calorie labelling and other obesity policies: a cross-sectional survey study of UK adults with eating disorders and other mental health conditions.

Raffoul, A., Gibbons, B., Boluk, K., Neiterman, E., Hammond, D., & Kirkpatrick, S. I. (2022). “Maybe a little bit of guilt isn’t so bad for the overall health of an individual”: a mixed-methods exploration of young adults’ experiences with calorie labelling. *BMC public health*, *22*(1), 938.

Roberto, C. A., Haynos, A. F., Schwartz, M. B., Brownell, K. D., & White, M. A. (2013). Calorie estimation accuracy and menu labeling perceptions among individuals with and without binge eating and/or purging disorders. *Eating and Weight Disorders-Studies on Anorexia, Bulimia and Obesity*, *18*, 255-261.

Robinson, E., Smith, J., & Jones, A. (2022). The effect of calorie and physical activity equivalent labelling of alcoholic drinks on drinking intentions in participants of higher and lower socioeconomic position: An experimental study. *British Journal of Health Psychology*, *27*(1), 30-49.

Seward, M. W., Block, J. P., & Chatterjee, A. (2018). Student experiences with traffic-light labels at college cafeterias: a mixed methods study. *Obesity science & practice*, *4*(2), 159-177.

Shoychet, G., Lowe, C. J., & Bodell, L. P. (2023). Does menu labelling influence food choice and consumption in female undergraduate students?. *Canadian Journal of Behavioural Science/Revue canadienne des sciences du comportement*, *55*(3), 220.

## Excluded studies at full text screening

Not Empirical Data: 9
(Daley & Bleich, 2021; DePaolo, 2018; Rodgers & Sonneville, 2018; Kaur et al., 2022, Llewellyn et al., 2023; Olsen, 2021; Seward et al., 2020; Whiteside, 2022; McGeown, 2019)

Not Out of Home Sector: 2
(Kesby et al., 2019; Ebneter, 2013)

No Data on Eating Disorders: 3
(Kim et al., 2022; Robinson et al., 2023; Roseman et al., 2023)

Review Article: 1

(Moghimi & Wiktorowicz, 2019)

Dissertation later published in peer review: 2
(Moore, 2014; Roberto, 2013)

Wrong Study Design: 1
(Lillico & Hammond, 2013)

## References for Excluded Studies

Daley, A. J., & Bleich, S. N. (2021). Should physical activity calorie equivalent (PACE) labelling be introduced on food labels and menus to reduce excessive calorie consumption? Issues and opportunities. *Preventive Medicine*, *153*, 106813.

DePaolo, A. (2018). Menu Calorie Labeling and Eating Disorders. Chicago, Illinois Academy of Nutrition & Dietetics 2018

Ebneter, D. S. (2013). *The effects of low-fat labeling and caloric information on food intake* (Doctoral dissertation, [Honolulu]:[University of Hawaii at Manoa],[December 2013]).

F. Rodgers, R., & Sonneville, K. (2018). Research for leveraging food policy in universal eating disorder prevention. *International Journal of Eating Disorders*, *51*(6), 503-506.

Kaur, A., Briggs, A., Adams, J., & Rayner, M. (2022). New calorie labelling regulations in England. *bmj*, *377*.

Kesby, A., Maguire, S., Vartanian, L. R., & Grisham, J. R. (2019). Intolerance of uncertainty and eating disorder behaviour: Piloting a consumption task in a non-clinical sample. *Journal of behavior therapy and experimental psychiatry*, *65*, 101492.

Kim, Y. H., Lee, S., & Barber, N. (2022). Food choice behavior: a case study in South Korea. *British Food Journal*, *124*(5), 1641-1658

Lillico, H., & Hammond, D. (2013). Calorie Labels on Menus: The Effect on People with Eating Disturbances. *Journal of Nutrition Education and Behavior*, *45*(4), S52-S53.

Llewellyn C.H., Sheen F., Conway R., Solmi F., Steptoe A., Nicholls D. (2023). Towards inclusive public health messaging for obesity and eating disorders: a focus on children and young people. *30th European Congress on Obesity (ECO 2023)*

McGeown, L. (2019). The calorie counter-intuitive effect of restaurant menu calorie labelling. *Canadian Journal of Public Health*, *110*, 816-820.

Moghimi, E., & Wiktorowicz, M. E. (2019). Regulating the fast-food landscape: Canadian news media representation of the healthy menu choices act. *International journal of environmental research and public health*, *16*(24), 4939.

Moore, K. (2014). *Individual differences in restrained eaters.* (Doctoral dissertation, Iowa State University)

Olsen, L. (2021). Calorie labels in restaurants: the impact on eating disorders. *bmj*, *373*.

Roberto, C. A. (2012). *Science-based Food Policy: Investigating Food Marketing, Menu Labeling and Front-of-package Food Labeling* (Doctoral dissertation, Yale University)

Robinson, E., Boyland, E., Christiansen, P., Haynos, A. F., Jones, A., Masic, U., ... & Marty, L. (2023). Is the effect of menu energy labelling on consumer behaviour equitable? A pooled analysis of twelve randomized control experiments. *Appetite*, *182*, 106451.

Roseman, M. G., Joung, H. W., Ossorio, E. M., & Valliant, M. (2023). College students’ attitude toward menu labeling differs by sex, BMI, weight management status, and athletic type. *Journal of American College Health*, *71*(1), 117-123.

Seward, M. W., & Soled, D. R. (2020). Unintended consequences in traffic-light food labeling: A call for mixed methods in public health research. *Journal of American College Health*, *68*(5), 465-467

Whiteside, A. (2022). Collateral damage in a reductionist strategy: the effect of calorie labelling on those with eating disorders. *BMJ*, *377*
